# Simple Syllabic Calls Accompany Discrete Behavior Patterns in Captive *Pteronotus parnellii*: An Illustration of the Motivation-Structure Hypothesis

**DOI:** 10.1100/2012/128695

**Published:** 2012-05-22

**Authors:** Matthew J. Clement, Jagmeet S. Kanwal

**Affiliations:** ^1^Laboratory for Auditory Communication and Cognition, Department of Physiology and Biophysics, Georgetown University Medical Center, 3900 Reservoir Road, NW, Washington, DC 20057-1460, USA; ^2^Department of Neurology, Georgetown University Medical Center, 3900 Reservoir Road, NW, Washington, DC 20057-1460, USA

## Abstract

Mustached bats, *Pteronotus parnellii*, are highly social and vocal. Individuals of this species roost in tight clusters, and emit an acoustically rich repertoire of calls whose behavioral significance is largely unknown. We recorded their social and vocal behaviors within a colony housed under semi-natural conditions. We also quantified the spatial spread of each bat's roosting location and discovered that this was relatively fixed and roughly confined to an individual's body width. The spatial precision in roosting was accompanied by an equally remarkable match between specific vocalizations and well-timed, discrete, identifiable postures/behaviors, as revealed by logistic regression analysis. The bodily behaviors included crouching, marking, yawning, nipping, flicking, fighting, kissing, inspecting, and fly-bys. Two echolocation-like calls were used to maintain spacing in the colony, two noisy broadband calls were emitted during fights, two tonal calls conveyed fear, and another tonal call signaled appeasement. Overall, the results establish that mustached bats exhibit complex social interactions common to other social mammals. The correspondence of relatively low frequency and noisy, broadband calls with aggression, and of tonal, high frequency calls with fear supports Morton's Motivation-Structure hypothesis, and establishes a link between motivation and the acoustic structure of social calls emitted by mustached bats.

## 1. Introduction

Nocturnal habits, relatively secure roosting locations, and the ability to fly and produce ultrasonic sounds have allowed many species of microchiropteran bats to evolve an extensive and sophisticated system of acoustic social communication without the fear of being detected by predators [1, 2]. In most instances, these same behavioral characteristics also make it difficult to study their audiovocal communication behavior. When such studies are possible, the tremendous diversity of species among microchiropteran bats can facilitate an analysis of acoustic signal design for audiovocal communication. 

Previous behavioral studies in bats have deciphered the vocalizations accompanying particular specialized behaviors, such as mother-infant interactions [3–7], copulation [8–10], sexual displays [11, 12], and various foraging activities [13–17]. Other studies have described and categorized the spectral structure of a large set of vocalizations without addressing their specific social functions [1, 18]. Data sets describing the acoustic structure of communication sounds and associated behaviors are available for only a few species, namely, little brown bats, *Myotis lucifugus* [19], Mexican free-tailed bats, *Tadarida brasiliensis mexicana *[20], leaf-nosed bats *Carollia perspicillata* [21], and false vampire bats, *Megaderma lyra* [22], as well as a few other Trinidadian bat species [23]. Even in these species, analysis was restricted to a few specific behaviors. Until recently, a statistical analysis of the data was commonly missing given the relative unavailability in the past of precision video and high speed audio capture and analysis methodologies.

**Figure 1:**
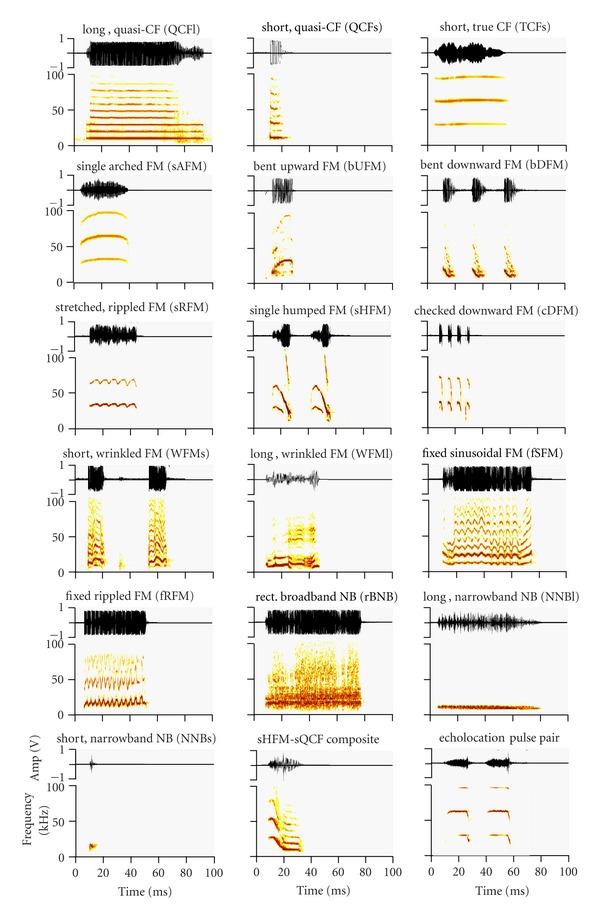
Amplitude envelopes (above) and spectrograms (below) of 16 different call types emitted by *P. parnellii. *An example of a composite of the single humped FM (sHFM) and short quasi-CF (QCFs) syllables, as well as a pair of echolocation pulses are also shown. All sounds were digitized at 250 kHz (sampling rate) and band-pass-filtered between 5 and 100 kHz. Spectrograms were produced using a fast Fourier transform (FFT) with a final time step of 1 ms. Amplitude range of frequency spectrum was 40 dB or higher. Call examples include sounds emitted by* P. p. parnellii *and* P. p. rubiginosus*. Figure adapted from [26].

Evaluating the possibility that spectral structures of vocalizations follow an evolutionarily stable acoustic design, as defined by Morton's Motivation-Structure hypothesis [24], requires both a large set of vocalizations and knowledge of a large number of behavioral contexts in which the calls are emitted. Mustached bats, *Pteronotus parnellii*, roost in large, mixed-sex clusters in caves throughout Central America [25]. Living in close proximity to conspecifics provides many opportunities for social interactions, especially when individuals roost within a tight cluster. Video recordings of individuals within a free-flying colony housed under seminatural conditions show that mustached bats have at least ten distinct and discrete behavioral interactions [26]. In addition to the physical interactions, individuals of this species produce at least 33 different types of vocalizations or “calls” for social communication [1]. These calls consist either of simple syllables or composites (a combination of two or more simple syllables without any intervening silent interval) that can be combined in a sequence (train) of similar syllables with short silent intervals. Simple syllables can be constant frequency (CF), frequency modulated (FM), or a noise burst (NB) type [1]. Acoustically uncluttered examples of 14 different simple syllabic calls are shown in Figure 1. Although the “phonetic-like” structural syntax in composites and trains of syllables has been studied at both the acoustic [1] and neurophysiological [27–29] levels, we know very little about species-specific behavior patterns and the call types associated with each behavior pattern. 

An analysis of social behaviors and call usage in mustached bats can be useful in addressing the evolutionary expansion of audiovocal communication in the specialized ecological niche of this species. Additionally, because of the discrete nature of mustached bat vocalizations, this analysis also provides an excellent opportunity to examine whether the acoustic signal structure in bat calls conforms to the rules of the Motivation-Structure hypothesis that is presumed to be widely applicable to avian and mammalian vocalizations [24, 30, 31]. Therefore, our objective was to investigate patterns of roosting positions and the behavioral context and social function of a variety of call types produced by the mustached bat, *Pteronotus parnellii*.

## 2. Methods

### 2.1. Animal Maintenance

Fifteen adult mustached bats, *P. parnellii*, were collected from a cave near Chaguanas, Trinidad, in September 2002. The ten males and five females were housed at Georgetown University and maintained at 28° to 30°C and 60% to 70% humidity under Biosafety Level II conditions with a 6 : 18 hour light-dark cycle. The colony was kept in a 4.0 m × 2.5 m × 2.5 m flight room where they could fly at will and roost in two upside-down pots fixed on the ceiling. The inside surface of the pots was coated with a 1 : 1 cement and Plaster-of-Paris mixture to provide a rough surface for roosting. The bats were provided mealworms and vitamin fortified water *ad libitum*.

### 2.2. Audio-Video Recordings

To establish associations between *P. parnellii* social calls and other behaviors, we made audio-video recordings of the bats with a Sony TRV310 digital HI8 video camera with an attached Optimus unidirectional condenser microphone (flat, within a 5 dB range; sampling rate of 44 kHz). We used a Lorex VQ-2120 infrared light so that we could record in the absence of visible light. We supplemented this with simultaneous ultrasonic recordings made with a bat detector (model U30; Ultrasound Advice), band-pass-filtered (between 4 and 100 kHz; model 3550; Krohn-Hite), digitized with a PCMCIA card (DAS16/330; Computer Boards, Inc.) at a sampling rate of 250 kHz for the broadband spectrum (flat with 5 dB up to 100 kHz), and recorded to a personal computer (Inspiron 7500; Dell Computers). A RACAL ST0705 tape recorder (set to 30 in/s) was also used to obtain high-resolution recordings of mustached bat calls for a general analysis of call structure. To reduce excess noise before recording, sound frequencies below 5 kHz and above 100 kHz were filtered out using a Krohn-Hite filter (model 3550) with a 24-dB/octave slope. A 20 dB Hewlett Packard 465A amplifier was used to magnify the oscilloscope trace and audibility of the band-passed frequencies. A two-channel Tektronix 2211 digital storage oscilloscope was used to compare the quality of the original and the recorded sounds. A minispeaker connected to the output of the amplifier was also used at times to monitor the bat vocalizations. The narrowband (low resolution) recordings were aligned to high-resolution broadband recordings to confirm call identity and were used to analyze the timing of vocalizations in relation to behaviors. Both high- and low-resolution digitized sounds were analyzed with SIGNAL software (version 3.0; Engineering Design) using a 512-point FFT and a Hanning window to produce spectrograms. Digital video was processed with Macintosh iMovie software.

Each bat was marked, either with a distinctively ornamented collar or by a distinctive bare skin pattern created by applying depilatory cream on the head. Since the bats spent almost all of their time inside the artificial roosts, we placed our camera 1.5 meters below the roost and directed it upward to focus on this small area. Although we lost sight of some individuals for short periods of time, the setup allowed us to make detailed behavioral observations on the roosting bats. Video recording sessions lasted for 15 to 25 minutes and occurred at various times of the day and night between April and October 2003.

### 2.3. Quantifying Roosting Position

To quantify the roosting positions of the bats, we took a photograph of the colony exactly five minutes into each recording session. Photographs were also taken overnight, and the task was automated using a programmable webcam (CS430, Intel, Inc.) with the included commercial software using a USB universal host controller interface. Photographs were transmitted in digital format over the internet and saved on a hard drive for further analysis. We recorded each bat's position in the roost and then mapped their locations relative to fixed points in the roost.

### 2.4. Scoring Social Behavior

Mustached bats perform a variety of distinct behaviors while roosting [26]. Each behavior is a discrete action, with a clear beginning and end. An occurrence or nonoccurrence of a behavioral event was scored as 1 or 0, respectively. As we reviewed the video, we recorded each behavior, its context, the bats involved, its start time, and duration. These behaviors included the following.

**(1) Crouching**

Both male and female mustached bats exhibited an upside-down crouch while hanging in the flight room or in a small cage. They slowly bent upwards and touched their nose to the substrate.

**(2) Marking**

In marking, a hanging bat thrust its hips forward and briefly rubbed its anogenital region against the substrate.

**(3) Grooming, Licking, and Yawning**

Grooming and licking were self-directed and were exhibited spontaneously in a resting state. In grooming, the bat hung from one foot and used its other foot to comb its fur and wing membranes. Grooming bats also opened either a wing or the tail membrane and cleaned the surface with the tongue. We only observed autogrooming, never allogrooming. The act of “yawning” occurred when the opening between the upper and lower jaws was at an obtuse angle.

**(4) Nipping**

A short, rapid snapping movement, involving the head and jaw, that was directed at a neighboring individual was classified as a “nip.”

**(5) Wing Flicking**

Wing flicking consisted of one to five rapid to-and-fro movements of a slightly open wing directed at another bat.

**(6) Boxing and Poking**

In boxing and poking, typically performed by two males, a bat thrust the digits of the forearm (folded wings) at another individual followed by a rapid withdrawal.

**(7) Wrestling and Biting**

During the “wrestling” behavior, bats briefly held each other with their flexed forearms and/or semiextended wings. Biting was identified when one bat clamped its jaws on another firmly, without the instant withdrawal that characterized nipping. In intense cases, nipping, flicking, boxing, wrestling, and biting could be combined during a fight between two or three bats.

**(8) Arching Back and “Kissing”**

Males typically performed the act of arching and “kissing” another individual. One male rapidly and repeatedly arched its back to contact the snout region of a neighboring male. The second male would often lick the face of the first male during this encounter.

**(9) Inspection**

During inspections, a male bent towards a female and brought his nose close to her genital region, presumably to detect pheromones and vaginal secretions. The female frequently aided his inspection by turning her hips towards him and slightly spreading her wings.

**(10) Fly-by**

During the fly-by behavior, a bat would fly into, out of, or past the mouth of the roost.

**(11) Other Behaviors**

Other behaviors consisted of shaking legs as if shivering, lateral body shifts, and upside-down walking a short distance (while hanging) within the roost or on the ceiling. However, these movements were too subtle and graded and their onset and offset too ambiguous to be scored in a consistent manner. Additional complex sequences of social behaviors, such as those associated with foraging, also could not be scored consistently.

### 2.5. Scoring Social Calls

Social calls in mustached bats consist of either simple syllables or “phrases” [1]. A syllable is defined as a discrete vocalization surrounded by periods of silence. A “phrase,” or simply a call, is defined as a series of syllables separated by less than 500 ms of silence. A phrase can include one or more syllables of one or more types. Mustached bats produce a complex suite of social calls (Figure 1). We classified each syllable based on the spectral criteria described in Kanwal et al., 1994 [1] for the *P. p. parnellii* subspecies. Syllables were named based on the geometrical shape of the spectrograms (e.g., rectangular, sinusoidal, etc.), and on whether the sound was frequency modulated (FM), constant frequency (CF), or a noise burst (NB). In addition, syllables were described as short if <50 ms and long if >50 ms in duration [1]. 

Many bat species emit echolocation pulses through their nasal cavities, and the vocal origin of all of their communication sounds is not certain. Other species, for example, *Saccopteryx bilineata*, *Myotis lucifugus*, and *Tadarida brasiliensis* are New World bats that, like mustached bats, emit echolocation pulses through the mouth [18–20, 23]. This allowed us to track both echolocation pulses and calls emitted by an individual. We assigned a call to a particular bat by matching its mouth, head, and body movements to the recorded sounds when only the observed bat was vocalizing and no other sounds were recorded. It was not always possible, however, to match a recorded call to a specific individual, such as when the vocalizing bat was not in the camera's field of view or when more than one vocalization was emitted simultaneously. We considered a call to be directed to a particular bat if the vocalizing bat turned its head towards that bat when calling. In a minority of cases, the call was not directed toward any particular bat. We scored each vocalization as a 1 and period of silence as 0.

### 2.6. Experimental Manipulations

We performed several experimental manipulations to elicit behaviors and vocalizations in order to better understand the relationships between social context, behavior, and vocalization. Over a period of several days, we isolated each bat in a small cage and gently poked it with a blunt object to mimic an agonistic interaction. We recorded the behaviors and vocalizations produced by the bat. We also isolated each bat and gently applied a single drop of water to its face to test the animal's vocal response. On five occasions, we randomly selected three males and three females from the colony and placed them in a cage. After allowing the bats to acclimate, we recorded one minute of behavior and vocalizations to quantify male-female differences. 

We also performed manipulations on bats in the flight room. We initiated a disturbance by having a researcher enter the flight room for one minute to stimulate the bats and increase social interactions. We recorded and scored social behaviors and vocalizations for ten minutes following the disturbance. We also removed seven males from the colony for several days to observe changes in roost position resulting from a change in colony composition. We then returned each male to the flight room to record the behaviors and vocalizations that accompanied their reintroduction.

### 2.7. Data Analysis

Our results rely on direct observations to establish associations between behaviors and vocalizations synchronized with movement of the jaws/mouth. The occurrence and nonoccurrence of a call and/or behavior was scored as a 1 and 0, respectively. Each call-behavior pair could then be scored as neither (0,0), both (1,1), call alone (1,0), or behavior alone (0,1). To score a (1,1), call onset must be separated from behavior onset by less than two seconds. In order to determine the significance of the relationships in the call-behavior pairs, we used the binary logistic regression function of Systat, version 10.0 (SPPS Inc). For each call type with at least 30 observations, we performed a series of multivariate regressions, with the behaviors as independent variables and one vocalization type as the dependent variable. From this, we obtained an odds ratio, which is the factor by which the odds of recording a vocalization type changes when a particular behavior is observed. In a logistic model, this value is more intuitive than the coefficient, which we calculated but do not present. We also report the likelihood ratio and McFadden's *rho*-squared statistic, which are roughly analogous to an F-test and an *R* ^2^ statistic, respectively. An estimate of the power of the model to predict vocalizations is given by the specificity measure. We also applied a Pearson chi-squared test to the logistic model fitted to the data. To evaluate the effect of a disturbance on the rate of inspections, we treated the inspection events as Poisson processes and used confidence intervals from Dowdy and Wearden [30]. We also used a two-sample *t*-test to test for changes in the rate of marking and crouching. Research on animals was performed in a humane manner, followed ASM guidelines, and was approved by the Animal Care and Use Committee of Georgetown University.

## 3. Results

### 3.1. Roosting Preference within a Colony

**Figure 2:**
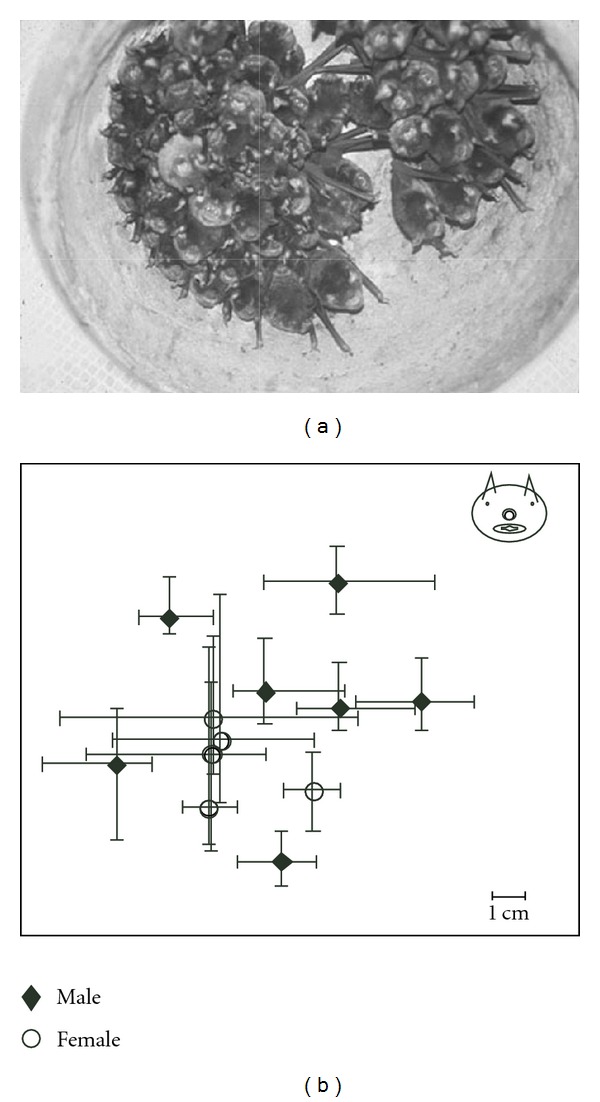
(a) A group of approximately 65 mustached bats roosting within an upside-down clay pot mounted in the ceiling of the flight room. (b) The range of roosting positions for mustached bats, over a two-week period, by sex. The bat's head (top right) is drawn to scale to indicate that the farthest extent of roosting location is typically the size of the body width of an individual. Symbols (solid diamonds for males and unfilled circles for females) are located at the mean (center point) location of an individual within the roost, and the bars indicate the range of individual-specific roosting locations.

In captivity, the bats roosted in a tight, mixed-sex cluster inside one of the artificial roosts (pots), although two were available. Due to the size of the room and the fixed location of their food source, the captive bats rarely left the roost. The roosting bats typically faced the outer edge of the roost (Figure 2(a)). Thus, most of the bats made a dorsal-to-ventral bodily contact, although bats in the center also made dorsal-to-dorsal contact. A ventral-to-ventral orientation was only seen during agonistic behavior. 

Based on photographs of the roosting bats, we found that the roosting patterns in mustached bats were very stable in the short term. They maintained the same location, relative to the roost and each other (Figure 2(b)). Across all individuals, the males stayed within an average area of just 6.1 cm^2^, whereas the females roamed across 19.5 cm^2^ (*t* = 2.25, *P* = 0.09). The males also commanded more exclusive space than females, sharing, on average an area of 1.0 cm^2^ with other males and 3.2 cm^2^ with females (*t* = 1.72, *P* = 0.12). The females shared an area of 5.0 cm^2^ with males and 16.4 cm^2^ with other females (*t* = 2.45, *P* = 0.05; two-tailed *t*-tests).

In our small colony of bats, three males always stayed outside the roost. When one of these satellite males entered the roost, other males attacked him until he retreated to the ceiling of the flight room. When seven males in the roost were removed from the colony, the satellite males joined the colony of females. When the seven males were reintroduced, the satellites males were excluded from the colony once again. The satellite males were not significantly smaller than the other males based on forearm length (*t* = 0.77, *P* > 0.5), weight (*t* = 0.16, *P* > 0.5), or a ratio of the two (*t* = 0.55, *P* > 0.5).

### 3.2. Simple Syllabic Calls Accompanying Discrete Behavior Patterns

We performed a frame-by-frame analysis of ~5 hours (302 minutes) out of a total of 35.4 hours of recorded video to quantify discrete social behaviors. Overall, the bats spent roughly 67% of their time resting and/or echolocating and 20% of their time grooming. The remaining 13% was spent interacting with other individuals. We did not observe any copulation, pregnancies, or births during this study. Our April to October observations did not include the January mating period, and this species is not known to successfully reproduce in captivity. Social calls were recorded almost exclusively during social interactions. When not performing discrete social behaviors, the bats spent less than 2% of their time emitting social calls. 

There was a close and highly specific association between the different call types and social behaviors in *P. parnellii*. The correlation coefficients and related statistical measures for each behavior and accompanying call type are listed in Table 1. A total of 1053 behavioral events were recorded. These included crouches: 88; marks: 56; yawns: 22; nips: 79; wing flicks: 65; fights: 62; head turns and kisses: 199; inspections: 189; fly-by behaviors: 293. A total of 801 of these events were accompanied by social calls. Vocalizations included ten syllables, although only eight had sufficient data for analysis (>30 observations): long quasi-CF (QCFl): 4349; rectangular broadband NB (rBNB): 373; fixed sinusoidal FM (fSFM): 33; bent, upward FM (bUFM): 35; short, true CF (TCFs): 17; short, narrowband NB (NNBs): 51; descending rippled FM (dRFM): 479; stretched rippled FM (sRFM): 502; short, wrinkled FM (WFMs): 2; long, narrowband NB (NNBl): 25. Overall, the call-to-behavior association was robust as indicated by the very high likelihood ratios and *ρ* ^2^ values, which ranged from high to very high, with the exception of the NNBs sound. Each call type exhibited a high value for the specificity measure and a Pearson chi-square of near unity. Some calls were produced almost every time the behavior occurred, whereas a few others were produced less consistently, perhaps because additional factors or contexts determine the reliability with which they could be triggered.

**Nonsocial Behaviors**

**Figure 3:**
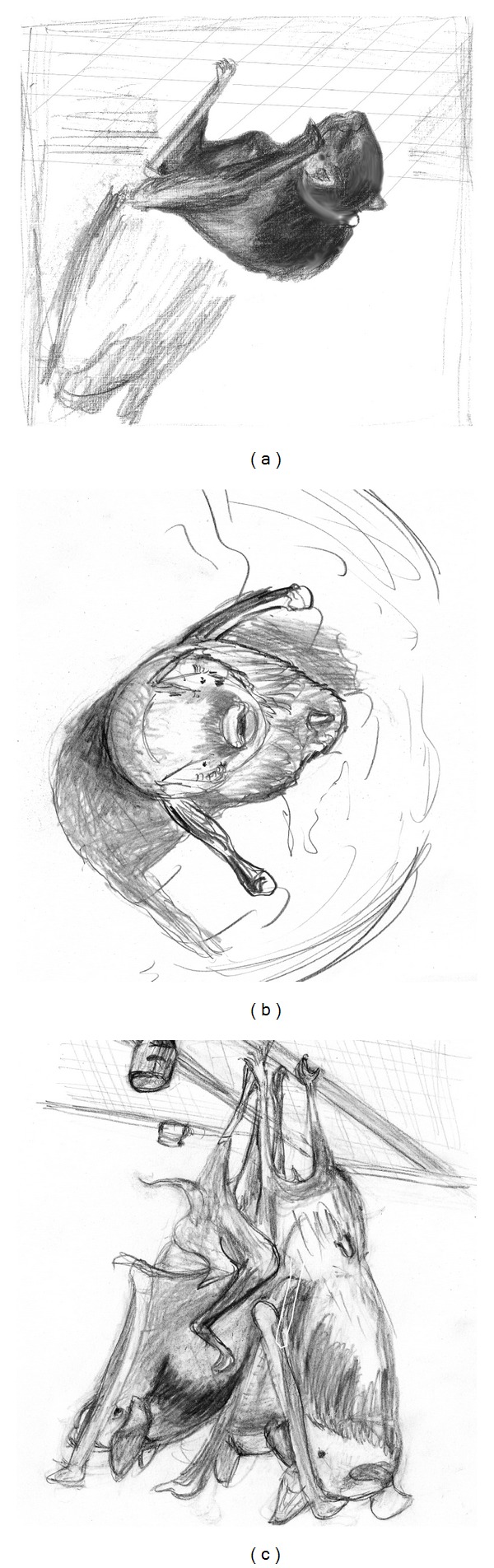
Drawings of individuals, traced from images acquired with an infrared camera showing common postures that are not typically associated with any vocalizations. Adapted from [26]. (a) Crouching. (b) Marking with hip thrust forward. (c) Three bats hanging with their backs in contact as the one on the left grooms it self.

**Figure 4:**
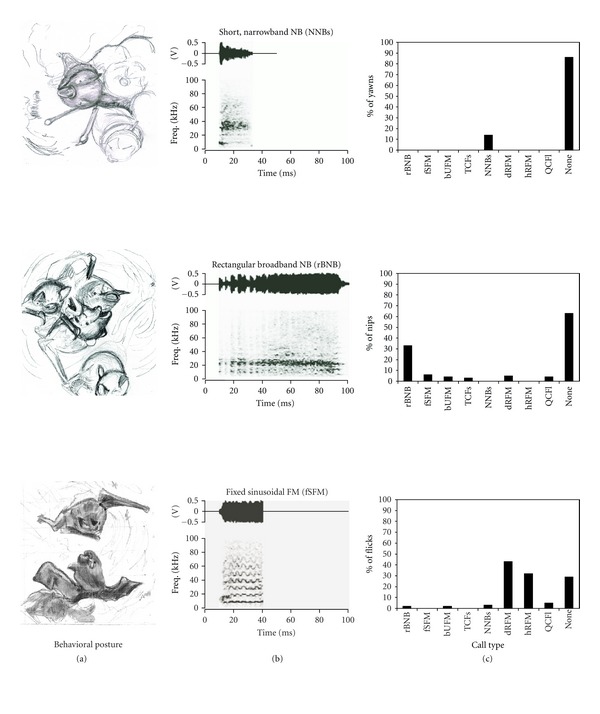
Association of social and vocal behavior in mustached bats. Left column shows sketches of common postures (left panel) and bar graphs (right column) to show the relative occurrence of different vocalizations associated with each behavior pattern. The middle panel shows amplitude envelopes (above) and spectrograms (below) of simple syllabic calls associated with each behavior. (a) Yawning. (b) Nipping. (c) Two bats flicking their wings at each other. Abbreviations: Freq. = frequency; V = volts.

The presence or absence of each call type was heavily dependent upon the behavior being concurrently expressed. Every sound, except the short, narrowband NB, was associated with one or more behaviors, and most behaviors were associated with at least one call type. No sounds were emitted during either crouching, marking, grooming or licking behaviors (Figures 3(a), 3(b), and 3(c)). Yawning behavior also was not associated with any sounds (Figure 4(a)). The short, narrowband noise burst (NNB) syllable represented just 2% of all syllables recorded. While 88% of NNBs appeared to arise spontaneously, 14% of yawns were accompanied by NNBs.

**Agonistic Interactions**

**Figure 5:**
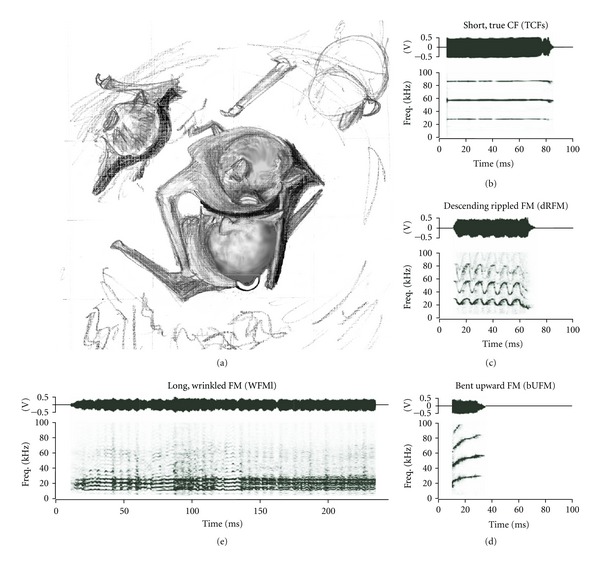
(a) Fighting among two males. (b), (c), and (d) Amplitude envelopes (above) and spectrograms (below) of associated simple syllabic calls. (e) A relatively long sequence of a composite consisting of long, wrinkled FM, broadband noise and sinusoidal FM type of simple syllabic elements. Abbreviations: Freq. = frequency; V = volts.

The agonistic behaviors, boxing and poking, elicited a similar set of simple syllabic call types (see Table 1). Noisy broadband “screech” call types, namely, rectangular broadband NB, fixed sinusoidal FM as well as bent, upward FM call types, were emitted during the agonistic behaviors of boxing, poking (Figures 4(b) and 4(c)), wrestling, and biting (Figure 5(a)). Although call types emitted during these behaviors were similar, wrestling and biting lasted longer, resulting in a greater number of calls. Of the 35 bUFM and 17 TCFs syllables that we recorded, 16 bUFM and 9 TCFs syllables occurred during fights. In addition to “screech-like” rippled FM sounds, wrestling and biting behavior was associated with high frequency tonal sounds such as the TCFs and the long, wrinkled FM (Figures 5(b) to 5(d)). The increased occurrence of the TCFs call type was significant only during boxing and poking. When a satellite male intruded into the roost, the nearest resident male would approach and often attack him. During this intrusion, the satellite male would emit long trains of long, quasi CF (QCFl) syllables. We observed 23 of these intrusions, during which the satellite male would emit long trains of QCFl syllables. Nipping represented a milder form of agonistic interaction than boxing, poking, wrestling, and biting and was not always associated with call production.

In total, we recorded rBNB on 118 occasions for 373 syllables and fSFM on 23 occasions for 33 syllables. Of 62 recorded fights, 45 included rBNB sounds, 5 had fSFM sounds, 10 had both, and only 2 had neither. Twenty-eight percent of the times that a bat was nipped it emitted an rBNB and 6% of the times it emitted a combination of rBNB and fSFM. We also recorded rBNB 26 other times and fSFM one other time, including some that seemed spontaneous and others that occurred during more common behaviors, such as a bat moving or shifting.

**Affiliative Social Behaviors**

**Figure 6:**
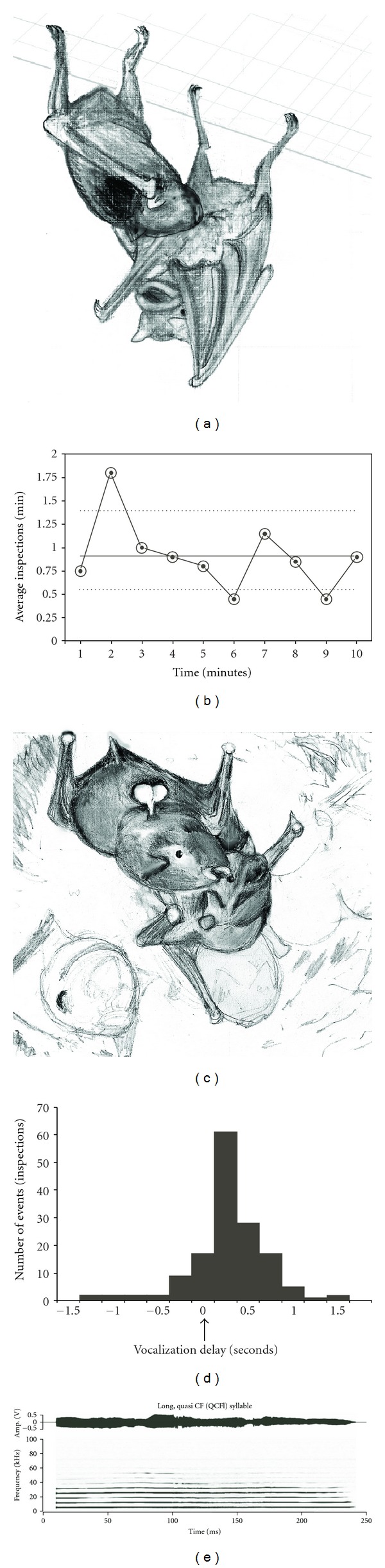
Appeasement behavior in mustached bats. (a) A male inspecting a female. (b) Inspection rate after a disturbance. The grey horizontal line shows the mean rate, and the dotted lines show the 95% confidence intervals. The rate is significantly higher in the second minute. (c) Two males “kissing.” (d) A histogram showing the temporal relationship of vocalization with the enactment of the kissing behavior. Zero time is indicated by the arrow and corresponds to the time of perceived contact of male's nose with the genital region of the female. (e) The amplitude envelope (above) and spectrogram (below) of a QCFl syllable.

Nonaggressive social behaviors were commonly associated with tonal syllables. We observed 177 “inspections” (Figure 6(a)); the inspection rate increased rapidly after a disturbance in the colony (Figure 6(b)). During inspections, onset of vocalization occurred within 0.5 s of behavior onset 74% of the time (mean = 0.13 s; SD = 0.67 s). We also observed 199 arching and kissing events (Figure 6(c)) in the 5 hours of recorded video. A long, quasi-CF call was emitted 171 times (86%) by the male while arching and kissing, and was commonly triggered by agonistic interactions between other individuals in the colony (Figure 6(d)). The 177 calls included 536 syllables, for an average of 3 syllables per call. The QCFl call, emitted during 174 inspections (98%), yielded 2154 syllables, and was usually of long duration and/or consisted of multiple syllables (Figure 6(e)). In a minority of cases, it was not possible to determine the caller. This difficulty was more frequent with short, soft, calls that were not associated with any actions. In a relatively rare event, we observed a male inspect another male 12 times, with the inspected male calling 6 times (50%) for 37 syllables. We did not see females inspect other bats.

**Warning Behaviors**

**Figure 7:**
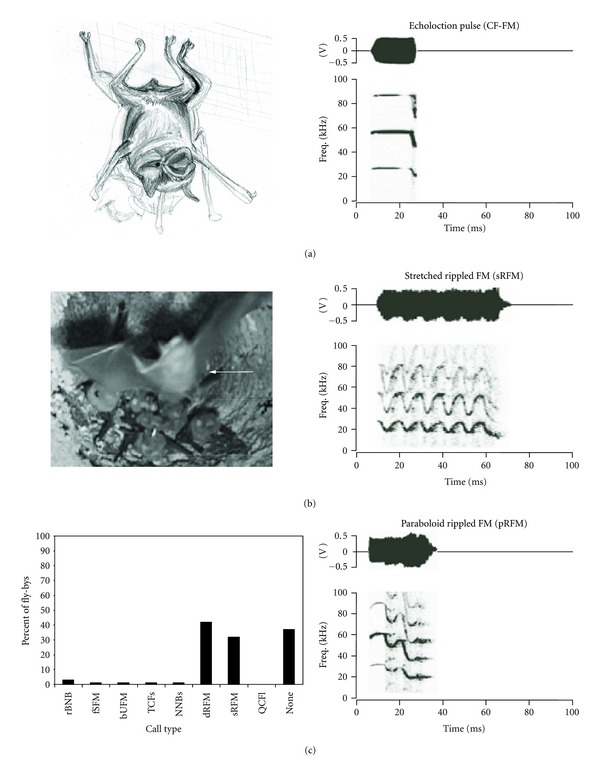
Echolocation and flying behavior in mustached bats. (a) A sketch showing the typical posture of roosting bat echolocating to inspect its surroundings. The amplitude envelope (above) and spectrogram (below) of the echolocation pulse are shown on the right. (b) A photograph of a bat (indicated by the white arrow) flying by a small cluster of bats roosting in an upside-down pot attached to the ceiling. Bats are viewed from below. Amplitude envelopes and spectrograms of the calls emitted during this behavior are shown in the two panels on the right. (c) A bar graph showing the frequency of occurrence of different call types during a fly-by.

Behaviors involving quick movements were frequently associated with syllables that appeared to be modified echolocation calls (Figure 7(a), left panel). During the locomotive behavior of a fly-by (Figure 7(b)), there was a small, but significant increase in the production of echolocation-like syllables. During fly-bys, nearby bats emitted stretched Rippled FM calls 158 times (223 syllables) and paraboloid upward FM-stretched rippled FM (pUFM-sRFM) composite calls 91 times (184 syllables) (right panel in Figures 7(b) and 7(c)). The stretched rippled FM call was structurally similar to a “buzz” of echolocation calls in rapid succession, but with an upward FM component in the intervening silence intervals (compare Figures 6(a) and 6(c)). Of the 293 fly-bys, the flying bat emitted these calls 177 times (60%). We also recorded these calls in 29 of 62 fights. A similar effect was observed for the same two calls during wing flicking, which was often accompanied by a noisy, fixed sinusoidal FM.

### 3.3. Manually Elicited Calls and Behaviors

We performed several manipulations to both confirm and explore the origin of different call types and the behavioral context in which they were emitted. For example, placement of water drops on a bat's nose resulted in immediate spitting and was frequently accompanied by the NNBs call type. In repeating this test with five of the bats, we recorded 113 NNBs sounds and only four other vocalizations. 

Poking a bat with a blunt probe elicited a few rBNB and fSFM syllables, but more often the bat sat still without vocalizing. In one instance, a poked bat responded with 33 single humped FM (sHFM) and 17 short, wrinkled FM (WFMs) in 30 seconds even though these calls were very rare in the flight room. Gently pinching the skin on the leg caused the bat to produce a wide gape together with the long wrinkled FM call type (see Figure 5(e)).

Immediately after a brief intrusion by a human visitor, the bats were agitated, echolocating constantly, moving about the roost, and sometimes flying away. In the second minute, as the bats' agitation wore off, inspections increased to a peak of 1.80 per minute compared to a ten-minute average of 0.91 inspections per minute (Figure 6(e)). Based on a Poisson distribution of inspection events, this peak rate represented a doubling of the rate of inspections and significantly higher compared to the average rate at a 95% confidence level [32].

Reintroducing a physically isolated bat back into the flight room increased the production of sRFM syllables and pUFM-sRFM composites. During the undisturbed period, these calls were used in just 12 of 43 fights (28%) and in 16 of 52 flights (31%). On the 9 days when we introduced a new bat, these syllables were used during 17 of 19 fights (89%) and in 161 of 241 flights (67%). We also found that when we held a male in a cage for a few days and then returned it to the colony, it significantly increased its crouching and marking from 0.27± 0.09 times a minute to 3.28 ± 0.72 times per minute in the first 10 minutes after rejoining the colony (*t* = 4.26, *P* = 0.048, *N* = 6).

### 3.4. Vocalization Frequency and Sex Differences

**Figure 8:**
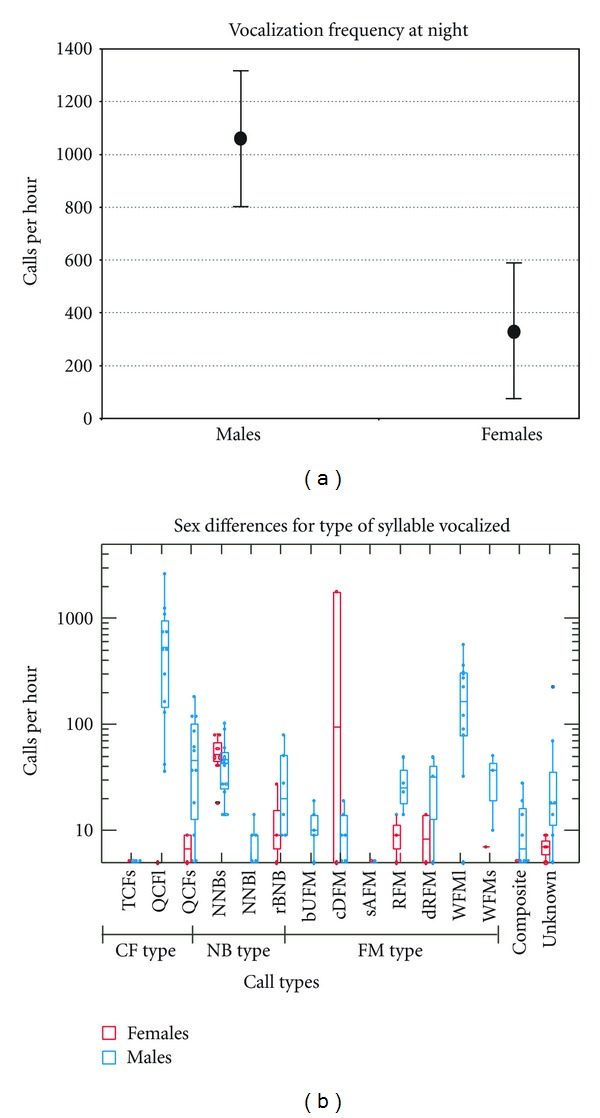
(a) Mean (solid circles) and standard deviation bars for frequency (number of calls per hour) of call production in males versus females. (b) Side-by-side box plots overlaid on density plots indicating the interquartile range (box edges) and the mean (dividing line within box) frequency of vocalization of different simple syllabic call types (CF, NB, and FM) in females (red) versus males (blue). Overlapping points, indicating the same value, are jittered. Composites are made of combinations of simple syllables. Whiskers at each end of the box indicate the 10th and 90th percentiles, and asterisks indicate data points outside this range. “Unknown” refers to call types that could not be easily classified. Other abbreviations are as in Figure 1.

For the randomly selected groups within males and females placed within the cage, sex differences in vocalization frequency were significant (*P* < 0.05; two-tailed, independent samples *t*-test; Figure 8(a)). We pooled the vocalizations of all 8 animals and examined the hourly rate of different call types in males versus females (Figure 8(b)). The long, quasi-CF syllables were the most common call type produced by either sex. Virtually all occurrences of the long, wrinkled FM call types were attributed to males, and those of the checked downward FM call type originated in females. The NNBs sound, noted above as a spitting sound, was produced in both sexes with equal frequency.

## 4. Discussion

### 4.1. Roosting Behavior in Mustached Bats

This study is the first to show that in mustached bats, individuals roost at highly restricted locations within a colony. Whether this is also true when individuals roost at locations outside the colony is less clear. The roosting locations of males overlap very little, at least in the short term. These roosting locations most likely drive several social interactions that take place in the dark where visual cues are absent, but olfactory and auditory cues are abundant. Conspecifics most likely maintain their territories by scent marks made by rubbing the anogenital region against the substrate. Accordingly, crouching is likely required to monitor the scent boundaries. In our observations, marking behavior sometimes alternated with crouching, which was consistent with the idea that these behaviors are related. Our observation that reintroduced bats increase their marking and crouching behavior is also evidence that these behaviors helped to establish and confirm roost position. Scent marking of territories with various exudates has been reported in several bat species [33, 34], including marking with the anogenital region for at least four species [20, 35].

### 4.2. Acoustic Signal Design in Mustached Bats

This study demonstrated a strong association between calls and discrete behavior patterns in the mustached bat (see Table 1). A tight temporal binding between calls and behaviors may be especially important for communication in species, such as mustached bats, that roost in a completely dark environment. Two noisy calls, rBNB and fSFM, and their composites were associated with two agonistic behaviors, nipping, and fighting. This association is consistent with the Motivation-Structure hypothesis, which posits that animals motivated by aggression will produce relatively low frequency, noisy, broadband calls [24, 31]. The level of noisiness in the fSFM call may correspond to the intensity of aggression, but this remains to be tested. In previous observations of mustached bats in a cage, playback of rBNB startled or warned an approaching bat and even made it turn back [26]. Other calls failed to elicit a similar response. 

In addition to the broadband calls, mustached bats occasionally produced two high frequency tonal calls during fights. The rapidly rising bUFM and the extremely high-pitched TCFs have structures typical of a fearful vocalization [24, 31, 36]. The acoustic structure of calls progresses from broadband types of calls that signal aggression to low-frequency tonal CF calls that are associated with appeasement behaviors (Figure 7). These data are in accordance with the Motivation-Structure hypothesis, which states that a tonal call is far more likely to indicate fear than aggression [30, 37]. A combination of CFs with an upward frequency sweep, as in a bUFM-TCFs composite, may indicate an intermediate state of fear and defiance. 

**Figure 9:**
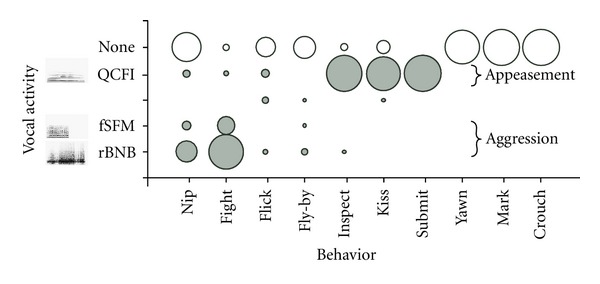
A bubble-plot (filled dark grey circles) showing the association of different sounds with different behavioral actions. The size of the bubble is proportional to the normalized (percentage of total events) frequency of occurrence of a call type. Per the Motivation-Structure hypothesis, which states that animals motivated by aggression produce relatively low-frequency, noisy, broadband calls (here illustrated by rBNB and fSFM calls) and those motivated by fear and/or appeasement produce tonal high frequency calls (here illustrated by QCFl calls). No calls were emitted during yawning, marking, and crouching. Fighting, which can be a complex and long-lasting behavior, produced the largest variety of call types. Unfilled circles are proportional to the percentage of the total number of events when a call did not accompany a particular behavior. Adapted from [26].

The long, quasi-CF (QCFl) call directed at an attacking bat is likely meant to appease as it mimics the cries of an infant, a common strategy in mammals [30]. This may be an indication of submission. Infant mustached bats commonly emit a QCFl-like syllable with a fundamental of ~10 kHz (Kanwal, unpublished observation, [38]) that may be used to appeal for food and/or attention. Females also emit the QCFl call during the inspection behavior, possibly a type of greeting behavior, in which the males sniff the genitals of females. Many terrestrial mammals use sniffing and genital inspection in greetings [30] including at least three species of bats [39]. When males “kiss” each other and emit the QCFl syllable, this is probably an appeasement call, used to foster affiliation and maintenance of peaceful relationships. Thus, variants of the QCFl call may signify different forms of appeasement, for example, to greet (peers: kissing), to appeal (infants), and to submit (outcasts/satellites), depending on the context and/or the social status of the emitter. Our data suggest that these appeasement calls may be considered as an extension of the Motivation-Structure hypothesis (see Figure 9). 

The descending and stretched rippled FM (dRFM and sRFM, resp.) syllables combine CF and FM components, much like the mustached bat's echolocation pulse, and appear to be used to maintain spacing. There is a striking similarity of structure and function between these calls and echolocation pulses, and they are emitted either to warn or in defiance of an approaching bat. Finally, one presumptive call, a short, narrowband noise burst, was shown to be associated with yawning and spitting behavior. Therefore, this sound may have been misclassified as a call as it appears to have little or no communicative significance. 

### 4.3. Acoustic Signal Design in Other Bat Species

The general pattern of call structure and function in mustached bats is largely consistent with empirical findings from other bat species. According to Pfalzer and Kusch [40], harsh broadband calls in bats are widely used during aggression, although buzzes and trills also fill this role. They also report that tonal calls in bats are used between mothers and pups, whereas more complex calls are used during mate attraction behavior. Both sexes of *Megaderma lyra*, an Old World microchiropteran species, use a low, multiharmonic “grumble” as an aggressive call, and males use a mix of tonal CF and FM calls in a display for females [22]. Male *Saccopteryx bilineata* use harsh, broadband calls to threaten other males and direct tonal calls towards females [41]. By our definition, an act or state of *appeasement* impacts positively on the affiliation between two individuals and may be triggered either spontaneously or because of an impending uncertainty. A state of aggression is a possible outcome of a state of defiance and warning and can also be triggered by other factors. Our data together with the work of Fenton [42] indicate that bat vocalizations generally follow Morton's (1977) predictions of signal design, which states that aggressive sounds should be of low frequency and noisy, whereas fear-related sounds should be of high frequency and tonal.

Similar to the sRFM in mustached bats, *Noctilio leporinus, Myotis volans,* and *Myotis lucifugus* produce a “honk” by adding a downward frequency sweep to their echolocation pulse [15, 19, 43]. *Pteropus poliocephalus* and* Carollia perspicillata* both use a “screech” to avoid collisions [21, 44]*. *Davidson and Wilkinson [41] found that a similar call, the screech-inverted-V, generally had no contextual association, and described the call to be a neutral notification “bark” for advertising territorial claims. A syllable with a classic inverted-V-shaped call structure, however, was not observed in mustached bats.

Except for composite calls that include the rBNB syllable, composites and complex sequences of calls were rarely observed during this study, and we are, at present, unable to assign a context or function to them. Since captive mustached bats do not mate or produce offspring, a similar study on wild populations of *P. parnellii* would almost certainly uncover new behaviors and vocalizations. Our results are a significant first step towards understanding audiovocal communication in a species that emits a complex echolocation pulse and employs a rich repertoire of calls for audiovocal communication.

## Figures and Tables

**Table 1:** A prediction success table generated from logistic regression of vocal activity against behavior (reproduced with permission from Cambridge University Press [26]). Numbers in the body of the table provide the classificatory power of the model and show how observations from each level of the dependent variable (call types) are allocated to predicted outcomes. The results provide an indication of the strong association of the different call types (columns) with specific behaviors (rows). The likelihood ratios are highly significant, and the specificity index ranges from 0.82 to 1.00.

Behavior/call	rBNB	fSFM	bUFM	TCFs	dRFM	sRFM	QCFl	NNBs
Crouch	0.0	0.0	0.0	0.0	0.0	0.0	0.0	0.0
Mark	0.0	0.0	0.0	0.0	0.0	0.0	0.0	0.0
Yawn	0.0	0.0	0.0	0.0	0.0	0.0	0.0	2.0
Flick	1.6	0.0	1.0	0.0	1.7*	2.4*	0.0	0.3
Box/poke	156.0**	313.9**	42.7**	34.6**	0.7	1.3	0.0	0.0
Bite	11.1**	63.9**	4.5*	4.5	0.2	0.0	0.0	0.0
Kiss	0.0	0.0	0.0	0.0	0.1	0.1	5.1**	0.1
Inspect-1	0.2	0.0	0.0	0.0	0.1	0.0	18.4**	0.0
Inspect-2	0.0	0.0	0.0	0.0	0.0	0.0	20.3*	0.0
Fly	0.6	1.7	0.5	0.9	2.4**	3.4**	0.0	0.1

Likelihood ratio	395.0**	111.0**	97.2**	58.6**	293.0**	250.0**	1084.0**	67.5**
rho^2^	0.48	0.46	0.36	0.31	0.20	0.22	0.52	0.17
Specificity	0.96	0.99	0.99	0.99	0.85	0.90	0.82	0.97
Pearson	1.00	1.00	1.00	1.00	0.99	1.00	0.82	1.00
